# Geminivirus-Derived Vectors as Tools for Functional Genomics

**DOI:** 10.3389/fmicb.2022.799345

**Published:** 2022-04-01

**Authors:** Bipasha Bhattacharjee, Vipin Hallan

**Affiliations:** ^1^Academy of Scientific and Innovative Research (AcSIR), Ghaziabad, India; ^2^Plant Virology Laboratory, Division of Biotechnology, CSIR-Institute of Himalayan Bioresource Technology, Palampur, India

**Keywords:** geminivirus, silencing system, crop improvement, CRISPR/Cas9, biopharmaceuticals, viral vector, peptide expression systems

## Abstract

A persistent issue in the agricultural sector worldwide is the intensive damage caused to crops by the geminivirus family of viruses. The diverse types of viruses, rapid virus evolution rate, and broad host range make this group of viruses one of the most devastating in nature, leading to millions of dollars’ worth of crop damage. Geminiviruses have a small genome and can be either monopartite or bipartite, with or without satellites. Their ability to independently replicate within the plant without integration into the host genome and the relatively easy handling make them excellent candidates for plant bioengineering. This aspect is of great importance as geminiviruses can act as natural nanoparticles in plants which can be utilized for a plethora of functions ranging from vaccine development systems to geminivirus-induced gene silencing (GIGS), through deconstructed viral vectors. Thus, the investigation of these plant viruses is pertinent to understanding their crucial roles in nature and subsequently utilizing them as beneficial tools in functional genomics. This review, therefore, highlights some of the characteristics of these viruses that can be deemed significant and the subsequent successful case studies for exploitation of these potentially significant pathogens for role mining in functional biology.

## Introduction

The “geminate” morphology which is typically a twinned capsid type of shape is the primary backdrop for the “geminivirus” nomenclature. Geminiviral genomes are termed ambisense because they comprise a coding segment which has two oppositely aligned reading frames (Hefferon, 2014). These viruses cause serious damage to crops resulting in having a devastating effect to produce quality and quantity globally (Navas-Castillo et al., 2011). Geminiviruses are characterized by their relatively small-sized genome and an extensive host range and can replicate in infected cells to enormously large copy numbers (Timmermans et al., 1994). They are categorized as class II viruses according to the Baltimore classification. This is the largest family of ssDNA plant viruses known in the virus kingdom, with newer variants constantly being discovered. Each paired particle encapsulates a single circular, ssDNA molecule that ranges in size from 2.5 to 4 kb contingent on the virus (Carrillo-Tripp et al., 2006; Yang et al., 2016). In the nucleus of the host cell, the genomic ssDNA is replicated by a rolling-circle process using double-stranded DNA (dsDNA) intermediates in a manner like the ssDNA bacteriophages. The International Committee on Virus Taxonomy (ICTV) has characterized the *Geminiviridae* family into fourteen genera, *viz*., *Becurtovirus*, *Begomovirus*, *Capulavirus*, *Curtovirus*, *Eragrovirus*, *Grablovirus*, *Mastrevirus*, *Topocuvirus*, *Turncurtovirus*, *Citlodavirus*, *Maldovirus*, *Mulcrilevirus*, *Opunvirus*, and *Topilevirus*, based on the vector (insect pest), host dissemination, genome organization, and pair sequence identities (Varsani et al., 2017; Zerbini et al., 2017; Fiallo-Olivé et al., 2021).

Understanding the distinct genomic components of geminiviruses is the primary step toward their genetic manipulation. The genome sequence typically contains a nonanucleotide “TAATATTAC” sequence, which is conserved, is in the form of a stem loop structure, and is important for the initiation of the rolling circle replication (RCR) (Stanley, 1995). The nonanucleotide is present in the DNA-A and DNA-B genomes of a bipartite geminivirus common region (CR) and a long intergenic region (LIR) for a monopartite geminivirus genome. Alpha and beta satellites are extra chromosomal small molecules which sometimes accompany certain old-world monopartite geminiviruses (Nawaz-ul-Rehman and Fauquet, 2009; Kumar et al., 2017; Gnanasekaran et al., 2019). A complete genome of a geminivirus would typically comprise CRs/IRs/SCRs (satellite-conserved regions) along with additional regulatory elements (Yang et al., 2016).

Insect vectors perform an imperative role in the introduction of these viruses within plant cells while keeping its primary target as differentiated cells (Kumar, 2019). Due to the non-existence of DNA replicase in these host insect cells, viral replication is seemingly improbable. Geminiviruses, therefore, instigate host cells to retrace the cell cycle from the dormant state to encourage recombination-dependent replication of the virus (Gutierrez, 1999). Insect vectors basically transmit geminiviruses through acquisition, retention, and ejection to host plant cells facilitating the entry of virions through the plant phloem sap. The interaction of the viral Rep protein with the whitefly’s proliferative cell nuclear antigen and DNA polymerase allows the TYLCV genome to replicate within the insect salivary glands (Pakkianathan et al., 2015). It is tempting to guess whether it can disrupt the insect cell cycle and produce an endoreplication cycle in the same way that plants can, or whether the insect has evolved alternative techniques. Because not all begomoviruses appear to reproduce in the whitefly, more research is needed to figure out why only some virus species replicate in the insect. Some begomoviruses can have active viral gene transcription within the insect.

Co-replication of the plant genome and the virion is initiated by geminiviruses through cell cycle reprogramming in the infected cells in order to compensate for its inability to produce enzymes for host cell replication and autonomous movement (Ramesh et al., 2017). Consequently, patterns of gene expression of the host are disrupted, necrosis pathways may get altered, and cell signaling and intercellular trafficking of macromolecules are redirected, causing dramatic changes in the host inherent environment. Additionally, geminiviruses modify host DNA methylation and miRNA pathways by encoding numerous suppressors of silencing, often causing plant developmental aberrations by suppressing multiple constituents of transcriptional and posttranscriptional gene silencing (Raja et al., 2010; Hanley-Bowdoin et al., 2013; Gnanasekaran et al., 2019; Prasad et al., 2019).

Typical geminiviral infection symptoms often include leaf curling, mosaic patterns of variegated colors, occasional vein swelling, and enations, often leading to the confirmation that cellular homeostasis and plant growth have been affected, thereby reflecting transcriptional changes. Geminiviral infectivity primes cellular signaling which leads to the misregulation of plant miRNAs concomitant to developmental shifts and hormone signaling. Although geminiviruses and their interactions with insect hosts have not been well understood, the latest studies have indicated that virus-mediated changes in defense and signaling pathways tend to occur. Past reviews have majorly focused on the pathogenicity of these viruses, their recombination, and synergism biology; hence, in this review, it is deemed appropriate to delve into the detailed understanding of utilization of these viruses as natural plant bioengineers. We have considered the already available information on the geminiviral genetic makeup and protein functions as well as the geminivirus–plant–insect tri-partnership in genetic manipulation. In this review, we have also focused on various essential characteristics of geminiviruses and have also extensively discussed the putative use of these significant pathogens for aspects ranging from functional biology to biopharmaceuticals.

## Geminiviruses Act as Deconstructed Vectors for Efficient Gene Transfer

Many geminiviruses, except for some begomoviruses, are usually not mechanically communicable as cloned DNA or virions (Inoue-Nagata et al., 2016). Certain *Agrobacterium* strains inhabit plant cells by transferring their T-DNA (transfer DNA) and cause proliferation and production of opine-like substances which the bacteria use as its carbon source (Moore et al., 1997). Initial studies conducted on *Agrobacterium tumefaciens* identified that the bacterium might be used for transferring infectious clones of *Cauliflower mosaic virus* (CaMV), and hence, the “agro-inoculation” or “agro-infection” technique was used as a sensitive assay to ascertain if transfer of DNA occurs during *Agrobacterium* inoculations of the geminivirus *Maize streak virus* (MSV) to a graminaceous plant, *Zea mays* (Howell et al., 1980; Grimsley et al., 1987). In fact, three *Mastrevirus* species have thoroughly contributed to replicon vectors, i.e., the monocot-infecting *Wheat dwarf virus* (WDV) and MSV and the dicot-infecting *Bean yellow dwarf virus* (BeYDV). Therefore, the first account of facile transmission of MSV was reported without involving leafhoppers to the maize crop. Despite the constant availability of newer isolates, *Mastreviruses* and *Begomoviruses* have remained the most popular taxa of the geminiviral replicon study (GVR) (Ellison et al., 2021). The study was considered a breakthrough of sorts as it confirmed that geminiviruses could be employed for development of transgenic cereals without the use of insect vectors, relying primarily on *Agrobacterium* transformation and their subsequent introduction to hosts (Grimsley et al., 1987). Biolistics and agroinfection have been commonly used to deliver engineered GVRs into plant cells, and many of these maintain mobility with plant tissue and insect cells allowing a transient system of expression to function efficiently. Precision-editing methods that need homology-directed repair (HDR) from a donor template molecule to integrate precise alterations into the genome benefit greatly from GVRs. The last decade has been watershed in exploratory studies between plant–geminivirus–whiteflies, although very few have explored the interacting proteins involved. Interactions mediated by plants between the geminivirus and insect vectors are important influences in understanding the biology of vector insects and the epidemiology of geminiviral diseases (Wang et al., 2012; Prasad et al., 2020).

Efficacious transfer of the T-DNA to juvenile embryos of MSV-infected germinated plants inferred the role of MSV as a highly sensitive genetic marker in maize–*Agrobacterium* pathogen–host interaction studies (Schlappi and Hohn, 1992; Escudero et al., 1996). Similarly, multiple experiments on WDV in wheat and barley showed the benefit of using geminiviral replication to evaluate direct gene transmission which lies in the context that transient expression could not show if cells were alive at the time of transfer of the gene. The successful infection of WDV in viable cells through *Agrobacterium* and microprojectile bombardment was used as a marker which methodically proved the efficacy of geminiviruses (Creissen et al., 1990; Chen and Dale, 1992).

**FIGURE 1:**
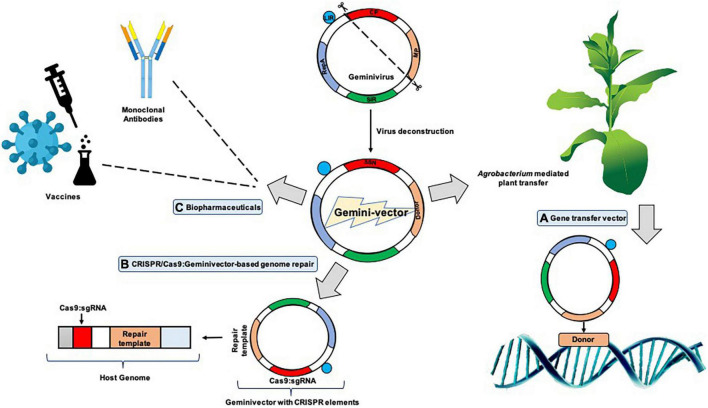
Geminiviral vectors and their functional relevance. Virulence factors like the viral movement protein (MP) and coat protein (CP) are detached to be replaced with the donor DNA and specific nucleases which give rise to “geminivectors.” These vectors have multidirectional functionalities in which they primarily act as **(A)** gene transfer vectors, where vectors are used for the transient expression of genes of interest, production of specific proteins, and participation and annotation of unknown gene characteristics, among others; **(B)** CRISPR/Cas9-based genome repair**:** where they are used for template repair in the host genome, while also essentially participating in genome editing; and **(C)** biopharmaceuticals: where production of important vaccines has been successfully conducted using geminivectors. Monoclonal antibodies have been prepared for the deadly *Ebola virus*, and antigen production for hepatitis B and HIV has been successful.

It was initially hypothesized that agroinfection with geminiviruses can potentially be used by random incorporation of viral DNA-carrying foreign genes into the plant chromosome to produce transgenic plants. This phenomenon is likely to occur rarely, as indicated by Bejarano et al. (1996) and Hanley-Bowdoin et al. (2013), which found proof of an ancient incorporation of geminiviruses into the *Nicotiana* genome. At that time, it was postulated that the geminiviruses usually did not get transmitted by seeds, which was indicative of certain mechanisms, which impedes them for germline invasion (Rosas-Díaz et al., 2017). However, very recent studies conducted had also reported accounts of seed transmission. TYLCV was the first detected begomovirus that was shown in tomato plantlets which had germinated from fruits produced by infected plants in 2013 and 2014 (Kil et al., 2016), in white soybean in 2016 (Kil et al., 2017), and in sweet pepper in 2018 (Kil et al., 2018). PCR studies revealed a complex relationship between the virus and seed tissues in this study; however, it could not be shown that the seed-accompanying virus causes tomato yellow leaf curl disease in plants grown from the infected seed. It is likely that plants infected with seed-borne inoculum have a lengthier dormant period, although this has yet to be experimentally verified. Furthermore, if TYLCV seed transmission is to a significant degree, there should ideally be more infected transplants grown in sheltered culture or in locations like in fields where the virus does not exist, particularly for hybrid seed-generated places where the virus does exist (e.g., China and Thailand). The intimate interaction of this virus with the seed during maturity was confirmed by the detection of TYLCV-IL replication in tomato and *N. benthamiana* flower reproductive organs. However, the considerable drop in TYLCV DNA load after surface disinfection of tomato seed implies that the virus is mostly found outside the seed coat as a contaminant (Pérez-Padilla et al., 2020), therefore laying to rest the debate as to whether the geminivirus is actually seed transmitted or not. Seed transmission of geminiviruses does not appear to be epidemiologically significant in the family so far. A more recent study has shown that this is a potentially significant attribute which can be relevant in the investigation for gene transfer systems as these viruses have a very broad-range host and are very pervasive in their infectivity. An exploration of geminivirus invasion in compromised plants showed that the virus did not infect the shoot apical meristem, apparently because of the high degree of cell division and the compounded lack of vasculature paths for the section (Lucy et al., 1996). This contrasted the study that had established that MSV could infect meristematic cells in the shoot apex by agro-inoculation (Grimsley et al., 1987). Past studies have proved that *Agrobacterium* transferred the T-DNA in the already preformed cells of the leaf primordia, hence clearing all doubts that geminiviruses indeed infected somatic tissues (Shen et al., 1992; Shen and Hohn, 1992, 1994). These findings laid down the foundation of understanding geminivirus infection and incursion that proved to be vital for the studies on manipulation of geminiviruses as vectors (Figure 1).

### Efficient Transient Expression Vector Systems

The transient expression procedure is one of the most convenient tools in molecular biology, giving appropriate results for further downstream processing as compared to stable transgenic development, which is both effort and time intensive. The expression of an extrinsic gene is simplified in this technique due to the negation of the host regeneration step, which makes the viral-mediated transient system very convenient to use while achieving the desired results. Host genome modification does not occur, and the geminiviral vectors can generate copious coding sequences to plants, posing as a competent alternative to stable line generation and exogenous gene manifestation (Yang et al., 2017).

The first proposal to construct a plant DNA virus backbone expression vector dates four decades back to the year 1978 (Hull, 1978). The geminivirus genome is conserved and already available in a plethora of online resources, and hence developing vectors with a geminiviral backbone is well established, although, in early reports, stable transformation (*Agrobacterium* mediated) and the plant virus-based vector usage were well thought out to be contending technologies. Lately, the merging of the two schools of thought brought the implication forward that agroinfection was the most effectual technique of application of “deconstructed” vectors to the host plant. This led to the rise of many groups of viruses like geminivirus, potexvirus, comovirus, and tobamoviruses for deconstructed viral vector development for rapid, enhanced levels of production of proteins in plants which could be a huge boon for the pharmaceutical industry (Xu et al., 2019; Figure 1).

The geminiviral genome structure knowledge is imperative in the understanding of vector preparation. This technique is very time efficient and requires small amounts of starting material. With the help of the Rep protein, geminiviruses autonomously multiply (Akbar Behjatnia et al., 1998). For initiation of RCR, replication-associated protein (Rep) binds to its complex binding site comprising the nonanucleotide-repeated sequence flanked by the transcription start sites (TSS) and the conserved TATA box in the case of begomoviruses. On the other hand, mastreviruses follow a slightly different mode of adherence as the Rep complex seemingly has a binding region juxtaposed upstream of two deviating TATA boxes and a replication start site downstream and at the bottom of the stem-loop conserved region (Fontes et al., 1992; Laufs et al., 1995; Castellano et al., 1999; Fondong, 2013). Among the first cases of introduction of geminiviral genomes in plants, the *Potato yellow mosaic geminivirus* (PYMV) was introduced within the Ti plasmid of *Agrobacterium* and agro-infection of *Nicotiana benthamiana*, tomato, and potato was successfully conducted (Buragohain et al., 1994). The approach engaged was that the head-to-tail dimeric PYMV on introduction to the Ti plasmid recombined in the IR region to form an autonomously replicating circular viral genome. The IR/CR contains the conserved nonanucleotide 5′-TAATATTAC-3′ sequence and a conserved stem loop structure within its origin of replication. This efficient strategy therefore became a conveniently implemented geminivirus vector assembly method (Peyret and Lomonossoff, 2015; Xu et al., 2019; Figure 1).

**FIGURE 2:**
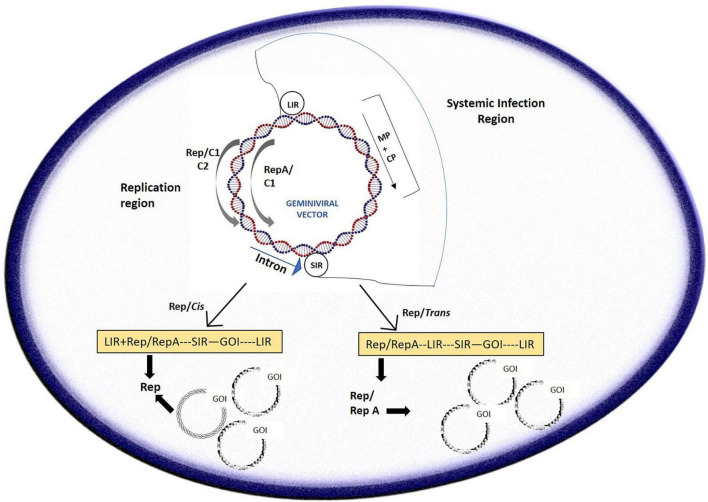
A typical geminivirus deconstruction vector: the LIR (long intergenic region) region initiates transcription of the movement/coat protein (MP/CP) regions while the SIR (short intergenic region) terminates transcription. Geminivirus-derived vectors can be sent to cells in two different pathways: the *cis* or autonomous and *trans* or tethered route wherein *cis* employs the Rep in its native position to the LIR, driven by a C-sense promoter, and can give rise to thousands of copies of the replicons and *trans* employs persistent production of Rep protein through stable integration to drive production of the replicon.

In other references, it was inferred that the process of the short/long intergenic region (SIR/LIR, respectively) recircularization by the viral *Rep/Rep A* proteins formed the crux of “LSL” vector (LIR–SIR–LIR) deployment (Mor et al., 2003; Figure 2). The case study of BeYDV consisting of the “LSL” sequence could be delivered within plants with successful replicon formation after recircularization. The Rep protein could be provided in *trans* which permits better regulation of the gene of interest, which is constructed on the LSL replicon, also allowing toxicity control of Rep. This system was also suggested to be fit for stable transgenic generation, with the gene of interest expressed from circular replicons on Rep gene induction which was then proven (Zhang and Mason, 2006). An LSL system was designed for stable tobacco cell cultures and potato lines based on the *Norwalk virus* (NV). With the Rep being under impact of the *Aspergillus nidulans* ethanol-inducible promoter, the gene of interest is constitutively expressed at reduced levels in the absence of ethanol. However, in the presence of ethanol, NV coat protein increased up to 10-fold in the cell cultures of tobacco and green fluorescent protein (GFP) got augmented up to 8 times (Zhang and Mason, 2006). These promising results were somewhat marred by the fact that silencing of the system eventually took place 8 days after induction in the transgenic potato lines; however, it could be resolved on adding a suppressor of silencing and additions of enhancers which could increase the amount of mRNA produced on addition and optimization of the amount of ethanol.

In a further improvement of the same system, the placement of the transgene was tweaked by having the *Tobacco etch virus* (TEV) 5′ UTR and soybean vspB 3′ region flanking it, aiming at the enhanced translational efficacy of the LSL (Zhang and Mason, 2006). When the promoter activity was weak and Rep/Rep A was made available in *cis* within the LSL, the transgene activity was at its highest (genes for reference were GFP, HBcAG, and NV coat protein). When a suppressor of silencing, TBSV p19, was added, the yield was further increased as the silencing machinery was severely affected (Huang et al., 2010), co-expressing two transgenes with the same T-DNA from a dual LSL construct which form two detached circular replicons individually overexpressing one transgene. The breakthrough of this system was that it was employed to generate 0.5 mg/g of anti-Ebola virus monoclonal IgG 6D8 utilizing the *Nicotiana benthamiana* system. On addition of the p19 suppressor of silencing in *cis*, instead of *trans* as was done till then, the expression system was treated as more efficient and by substituting the TEV 5′ leader with the “hyper translatable” *Cowpea mosaic virus* (CPMV)-based CPMV-*HT* 5′ leader (Sainsbury and Lomonossoff, 2008). The user-friendly BeYDV vectors pBYR1 and pBYR2 were then developed containing an extra kanamycin cassette, and the pBYR1p19 and pBYR2p19 variants had the p19 cassette instead of kanamycin (Chen et al., 2011).

The BeYDV-based pRIC system is another improved geminiviral vector system (Regnard et al., 2010). It is based on the pTRAC system which employs the tobacco Rb7 gene scaffold attachment area (Maclean et al., 2007) for a heightened heterologous gene expression (Halweg et al., 2005). This system was used to overexpress the *Human papillomavirus* CP (HPV-CP) and HIV CPp24 at 0.55 mg/g FWT and 3.23 μg/g FWT, respectively. The system has reported a replication efficiency up to a 1,000-fold and a protein expression fold change up to 7 times, indicating that this is a robust system of overproduction of antibodies and vaccines (Maclean et al., 2007). The *Beet curly top virus* (BCTV) which comprises a *Cassava vein mosaic virus* promoter (CsVMV) has also been employed to synthesize vaccines like *Hepatitis A-virus* VP1 protein in modest levels in fusion with IgG (Kim et al., 2007; Chung et al., 2011). With unique techniques like precise target-based editing and exploration of current cas9 homologs (C2c1, C2c2, C2c3, and Cpf1) (Lu and Zhu, 2017; Nakade et al., 2017), geminivirus vectors stand a lot to gain and get developed further (Peyret and Lomonossoff, 2015). Vectors were constructed using truncated regions of *Tomato leaf curl virus* (ToLCV), and it was deduced that a segment between CR and AC3 could replicate efficiently. Therefore, a viral vector was constructed which efficiently produced siRNAs, giving rise to a system that could be an efficient GIGS vector in a broad range of plants (Pandey et al., 2009).

Plant geminivirus-based replicating expression vectors have been widely employed in recent decades to improve the effectiveness of plant transient expression. The BeYDV-derived vectors have been successfully employed to explore the properties of phytohormones like α-naphthalene acetic acid, 6-benzyladenine, and gibberellins_3_ on plant biomass and efficiency using the GIGS process of transient expression in *N. benthamiana* L. leaves (Li et al., 2021). The transient enhanced expression *in planta* may provide an understanding of how to exploit plant growth regulators (PGRs) to recover the production of recombinant protein using geminiviral vectors as the expression source of desired genes.

There is a major potential for human gene expression studies in plants to showcase the biological activity in a safer system with easier accessibility to experimental plants and tissues. A recent study where a human vascular endothelial growth factor (VEGF) was transiently expressed in *Nicotiana benthamiana* showed that VEGF could be expressed in high levels and did not exhibit any cytotoxic effect on HaCaT (human keratinocyte) cells, while also inducing cell migration *in vitro*. VEGF is a very important factor which is pro-angiogenic and important for wound healing. The recombinant clone of the VEGF gene was made using the geminiviral vector pBYR2e, which indicated that the functional VEGF protein could be made in plants that can possibly be exploited for their use in tissue engineering and dermo-cosmetics (Bulaon et al., 2020). Using *Agrobacterium*-mediated transformation, the capacity of the DNA geminiviral vector with Rep-mediated replication to produce recombinant proteins transiently in aquatic microalgal species was tested in *Chlamydomonas reinhardtii* and *Chlorella vulgaris*. Representative antigen and growth factor proteins, the SARS-CoV-2 receptor-binding domain (RBD) and basic fibroblast growth factor (bFGF), were cloned in a geminiviral vector and employed for rapid transformation to express the proteins in a transient manner in *C. reinhardtii* and *C. vulgaris*, respectively. The outcome demonstrated that the geminiviral vector could allow the expression of both the proteins in both the algal species, with yields of up to 1.14 g/g RBD and 1.61 ng/g FGF in *C. vulgaris* and 1.61 g/g RBD and 1.025 ng/g FGF in *C. reinhardtii* at 48 h post transformation. As a result, this study showed that DNA viral vectors could be used to produce recombinant proteins in a simple, quick, and effective manner, overcoming the challenges of genetic transformation in these unicellular green microalgae. This notion brings up the possibility of researching and optimizing green microalgae as a cost-effective platform for producing medicinal and industrially important recombinant proteins in shorter time frames with higher yields (Malla et al., 2021). Transient expression methods are ideal for swift and versatile development and production of vaccines against viruses like 2019-nCoV that exhibit abrupt and unpredictable outbreaks due to their rapid and high-level protein production potential. Plants have been offered as a viable, cost-effective, efficient, ethical, and sustainable expression system, despite the fact that many recombinant proteins are created by microbial or mammalian cell-based expression systems. The introduction and refinement of transient expression systems have resulted in a significant reduction in protein synthesis time and an increase in recombinant protein yield in plants (Nosaki et al., 2021). Such case studies are the need of the hour, and having results which can impact the human life indirectly and directly shows that the there is a huge unexplored territory of viral deconstruction and manipulation which should be researched upon. Geminiviruses have, therefore, tremendous potential to be utilized as vectors for gene transfer, protein expression, and mutagenesis studies due to its highly efficient ssDNA genome and ability to mold under any exogenous deconstruction (Figure 1).

### Efficient Gene Silencing Vector Systems

The most common geminiviral silencing vectors initially developed were the begomoviral vectors derived from the following viruses: *African cassava mosaic virus* (ACMV), *Cabbage leaf curl virus* (CaLCuV), *Pepper huasteco yellow vein virus* (PHYVV), *Tomato golden mosaic virus* (TGMV), and *Tomato yellow leaf curl china virus* (TYLCCV) (Table 1). ACMV has been utilized to construct a functional reverse genetic tool by incorporating a partial *NtSu* gene (*Nicotiana tabacum sulfur gene*) which includes one unit of the magnesium chelate chloroplast enzyme (Fofana et al., 2004). This system was used for successful silencing of the orthologous gene of cassava resulting in the characteristic white and yellow dotted phenotype. Another gene of the same plant, *CYP79D2*, was cloned as a partial fragment in the ACMV vector which directly led to their downregulation, leading to a 76% lesser production of linamarin at 21 dpi, as the gene is one of the essential enzymes for the synthesis of linamarin in cassava. As the cassava transformation is a very laborious procedure, the ACMV-based gene silencing is very effective and can be used as a substitute to the stable transgenic approach (Fofana et al., 2004; Table 1). The bipartite CaLCuV opened new dimensions of silencing of singular or multiple endogenous genes in the entirely sequenced easy-to-handle model plant, *Arabidopsis thaliana.* The DNA A and B components have been utilized for vector development, resulting in extensive silencing patterns in the plants with the DNA A-modified vector. With the success of endogenous gene silencing or geminivirus-induced gene silencing (GIGS), this vector offers a distinct advantage of direct plant inoculation, bypassing the agro-transformation method, and could be used to augment phenotypes, for identification of redundant functions, and for understanding epistasis biology (Turnage et al., 2002).

**TABLE 1:** Examples of gemini vectors designed for expression/silencing and biopharmaceutical development.

Virus or satellite	Final outcome	Host	Gene target	References
African cassava mosaic virus	Silencing	*N. benthamiana, M. esculenta*	Su, PDS	Fofana et al., 2004
Cabbage leaf curl virus	Silencing	*A. thaliana*	Su, PDS	Muangsan and Robertson, 2004
Pepper huasteco yellow vein virus	Silencing	*C. annuum N. tabacum L. esculentum*	Su, Comt pAmt, Kas	Tao and Zhou, 2004
Tomato golden mosaic virus	Silencing	*N. benthamiana*	Su, PCNA	Kjemtrup et al., 1998; Peele et al., 2001
Tomato yellow leaf curl China virus DNA-β	Silencing	*N. glutinosa N. tabacum L. esculentum*	PCNA, PDS, Su	del Rosario Abraham-Juárez et al., 2008
Bean yellow dwarf virus	Expression/Silencing/Vaccine production/Monoclonal antibodies	*S. lycopersicum S. tuberosum N. tabacum*	ANT1, NPTII ALS1, ALS2, SEB vaccine, Ebola, HPV Ab	Baltes et al., 2014; Butler et al., 2015, 2016; Čermák et al., 2015
Wheat dwarf virus	Gene transfer/Expression	*T. aestivum, O. sativa H. vulgare*	Ubi, MLO, GFP Gus	Gil-Humanes et al., 2017; Wang et al., 2017
Chili leaf curl virus	Phloem-specific silencing/expression	*N. benthamiana*	eGFP, PDS	Kushwaha and Chakraborty, 2017
Cotton leaf crumple virus	Silencing	*G. hirsutum*	ChlI, PDS	Tuttle et al., 2012
Bhendi yellow vein mosaic virus β DNA	Silencing	*N. benthamiana*	Su, PDS, PCNA, and AGO1	Jeyabharathy et al., 2015

*The abbreviations are as such: Su, sulfur allele of magnesium chelatase complex; PDS, phytoene desaturase synthase; Kas, keto-acyl ACP synthase gene; pAmt, possible aminotransferase gene; Comt, caffeic acid O-methyltransferase gene; ALS1, acetolactate synthase 1; GFP, green fluorescent protein; NPTII, promoter of GUS and neomycin phosphotransferase; PCNA, proliferating cell nuclear antigen. These highlight the diverse roles of deconstructed vectors in functional genomics.*

Like CaLCuV, TGMV-derived vectors were also tested for DNA A/B efficacy, with DNA B showing better silencing efficiency. The TGMV B vector was specialized to use endogenous fragments <100 bp, thereby showing improved specificity which could also be tissue specific. Magnesium chelatase and proliferating cell nuclear antigen (PCNA) were downregulated all over meristematic tissues which showed that two different genes could be silenced simultaneously using the same viral vector components, using the immune-localization technique (Peele et al., 2001; Table 1). The PHYVV-derived vector was used to silence three gene fragments of *Comt* (coding for a caffeic acid *O*-methyl transferase), p*Amt* (a possible aminotransferase), and *Kas* (a β-keto-acyl-[acyl-carrier-protein] synthase) genes, resulting in a non-pungent chili pepper and was confirmed by assessing the reduced mRNA levels of individual genes and because of the heightened siRNA presence (Table 1). A unique characteristic of silencing was that fruiting was normal, although PHYVV-p*Amt* infections were asymptomatic. Non-pungent chili peppers could be efficaciously produced using this technique (del Rosario Abraham-Juárez et al., 2008). The TYLCCV Y10 isolate has an accompanying β satellite which has a dependency on DNA A for replication and encapsulation and is indispensable for symptom development in host plants (Tao and Zhou, 2004). DNAβ was modified to DNAmβ by replacing its C1 ORF with an MCS, effectively transforming it into a silencing vector. This resulted in a symptomless virus invasion with clear demarcated transgene-specific symptoms visible for inference. Multiple plants like *N. glutinosa*, *N. tabacum*, and *Lycopersicon esculentum* were successfully tested with the vector with multiple endogenous gene downregulation like PCNA, phytoene desaturase synthase (PDS), and GFP.

The phytoene desaturase (PDS) gene was chosen as an efficient visual indication of VIGS and was used to improve the VIGS system in cabbage utilizing the *Tobacco rattle virus* (TRV)-based pTYs and CaLCuV-based gene-silencing vectors (PCVA/PCVB). In response to pTYs and CaLCuV, the latter came out to be a more efficient system in terms of operation costs and expense because the pTY mode of VIGS requires the particle bombardment mode of plants using expensive instrumentation. CaLCuV-based GIGS involved the use of *Agrobacterium* and was relatively inexpensive and had a higher batch efficiency target. This geminiviral vector system has also been said to be equally efficient in other *Brassica* species, *B. rapa*, and *B. nigra*, and therefore a potent functional genomic tool for crop plants (Xiao et al., 2020).

Geminiviruses have a compact genome that makes cloning and sequencing simple, and propagation is significantly easier than *in vitro* RNA synthesis (Abrahamian et al., 2020). Furthermore, DNA is much less labile than RNA and could be inoculated directly. Moreover, because DNA vectors lack an RNA intermediary and so are not susceptible to RNA degradation, they have demonstrated to be more stable than RNA vectors. As a result, even if the transcripts are suppressed, the replicons continue to increase and transport, albeit at a comparatively slower rate than wild-type counterparts. This, consequently, was modeled as a powerful tool for functional genomic approaches and gene discovery (Table 1).

### Geminiviral Vectors and Genome Editing (GE)

Popular RNA vectors such as the *Potato virus X* (PVX) and TRV are commonly used for gene targeting and functional validation studies by transient silencing/overexpression of plant genes. Although effective, the insert size needs to be very small for RNA interference to occur (Dinesh-Kumar et al., 2003). The newer-generation genome-editing tools like CRISPR/Cas9 involve Cas9 delivery to plants, which is a large-sized protein and hence not appropriate for the RNA vector systems. Geminiviral replicons which are mutated to remove the infectivity have been developed to retain bigger-sized sequences like sequence-specific nucleases (SSNs) and DNA repair templates. BeYDV geminiviruses have been successfully proposed as CRISPR/CAS candidates for targeted genome editing and mutagenesis due to the zinc finger nucleases (Baltes et al., 2014). The advancement of viral modulation studies gave rise to the BeYDV deconstruction (Baltes et al., 2014) with it comprising the Rep/RepA-LIR-SIR components. The Rep A protein interacts with components of the cell cycle relating to replication while the Rep protein binds to the LIR region, starting a nick formation to initiate RCR, which is an important aspect for utilization for multi-copy generation of the donor and stimulate homologous recombination between donor and target. Being a very host-diverse system, BeYDV has been used (Čermák et al., 2015) to replace the endogenous anthocyanin constitutive promoter with a strong promoter, aiding as a marker for gene targeting at a very early calli stage in tomato, having an approximately 12-fold higher expression as compared to the routine *Agrobacterium*-T DNA delivery system. It was also used for creating targeted mutations in cassava as well as in potato (Butler et al., 2015, 2016; Hummel et al., 2018; Table 1). WDV and ToLCV are other geminiviruses which have been used for gene targeting in wheat by multiplying gene targeting (GT) efficiency manifold and achieving multifold targeted reporter gene integration in the hexaploid wheat genome (Table 1). WDV has also been successfully used in rice (Gil-Humanes et al., 2017; Wang et al., 2017). WDV was characterized and optimized by comparative replicon assessment to make it a suitable candidate for GT in cereal crops and for delivering CRISPR/Cas9 donor templates (Figure 1).

The above studies showed that GT could be successfully undertaken and had more reliability as compared to agro-transformation procedures but posed few challenges like low efficiency and high reliance on selectable or reporter exogenous markers like GFP and others. The CRISPR/Cas9-coupled geminiviral vector experimentations opened newer insights toward GT and editing (Figure 1). On BeYDV infection, *N. benthamiana* constitutively expressing Cas9 components showed relatively lower viral load and obstructed (or reduced) symptoms. BeYDV-based replicons were used for transient assays which detected Cas9 reagent-induced mutations within the virome, effectively reducing the virus copy number (Baltes et al., 2015). Ji et al. (2015) also worked out transient detections on *N. benthamiana* which comprised the Cas9-sgRNA construct which inhibited *Beet severe curly top virus* (BSCTV) accumulation and mutagenesis at targeted sequences, while overexpression of the same construct in transgenic *Arabidopsis* and *N. benthamiana* made the plants highly resistant to virus infection. Targeted coding sequence mutations in various geminiviruses caused the generation of viral variants adept in replication and systemic spread while noncoding IR mutations within geminiviruses provided viral intervention activity which limits significantly the replication and infectious variant generation, leading to long-lasting crop protection strategies against plant viruses (Ali et al., 2016).

A novel insight toward the use of geminiviral vectors was presented as the virus-induced gene editing (VIGE) protocol which is basically a geminiviral-centered guide RNA delivery system for plant genetic engineering using CRISPR/Cas9 (Yin et al., 2001; Yin et al., 2015). Cas9 is overexpressed in model plants like *N. benthamiana*, and geminiviral vectors comprising single-guide RNA (sgRNA) are transiently expressed targeting the gene of interest. This model has been touted to be used for generation of knockout libraries and has the potential to revolutionize crop genetic engineering (GE) in future. Recently, geminiviral reporter vectors WDV-GFP and WDV-GUS were constructed to deliver profuse amount of DNA to rice cells to make a practical KI (knock-in) method by combining CRISPR/Cas9 to produce double-strand breaks, with a noticeable 19.4% increase in KI frequency (Wang et al., 2017). Similarly, a geminiviral replicon (GVR) was used to deploy SSNs targeting the potato ALS1 gene, which resulted in a significantly lowered herbicide susceptibility phenotype. This validated the geminivirus-based CRISPR/Cas9 delivery approach to plant species and a novel GT methodology for vegetative propagated species (Butler et al., 2016), along with the increase in efficiency of homology-directed repair (HDR) and the possibility of stable transgenic line generation. HDR using a donor template molecule is often used to accomplish precise GT, which includes site-specific sequence changes and targeted insertions. SSNs such as zinc-finger nucleases (ZFNs) (Townsend et al., 2009), TAL-effector nucleases (TALENs) (Zhang et al., 2013), and CRISPR/Cas nucleases have been employed to induce HDR and restore stable GT events because induction of double-stranded breaks (DSBs) considerably enhances the frequency of HDR in plant cells (Puchta et al., 1993). Mutagenesis is the most common result of SSN-mediated DSB induction, although GT events are generally an order of magnitude less common. This is due to the prevalence of alternative DNA repair mechanisms, including as microhomology-mediated end joining and quasi end joining, as well as the necessity for HDR that DSB induction be synchronized with the donor molecule.

The ability of GVRs to efficiently reproduce template DNA, providing an adequate supply of HDR templates, is an advantage of GVRs in GT. However, geminiviruses’ reliance on the replication initiator protein (Rep) restricts their usage in gene editing because Reps are recognized to interact with a number of host cell proteins involved in the cell cycle, DNA replication and restoration, and DNA methylation (Maio et al., 2020; Kujur et al., 2021). Off-target genomic alterations are a serious problem linked with a constitutive overexpression of CRISPR/Cas9 systems. Ji et al. (2018) and Rato et al. (2021) created a virus-inducible method based on the promoter segments of the geminivirus BSCTV to overcome this limitation. The modified *Arabidopsis* producing virus-inducible Cas9 and gRNAs directly targeting the Rep gene were infected with BSCTV after validation in transient *N. benthamiana*. Plants had a very low virus load and displayed no symptoms. Crucially, there were no off-target genomic alterations in these plants (Ji et al., 2018). Another study described a Cas9 expression system controlled by a virus-inducible endogenous rgsCaM tomato promoter, which raised similar difficulties. Transient experiments validated the inducible expression of Cas9 in the *S. lycopersicum* when infected with the *Tomato yellow leaf curl virus* (TYLCV), as well as the decrease of Cas9 expression in tomato when infected with TYLCV (Ghorbani Faal et al., 2020). With viruses being highly mutating and failure of pest sprays in targeting viral infections and the viruses’ quick spread, genome editing has become a precision-based technology which could address the huge demand of targeted viral disease management in crop plants. GE is hence a critical tool for ensuring long-term food safety and security, as well as mitigating the problems faced by the world’s expanding population and climate change.

## Geminiviruses in Biopharmaceuticals

Geminiviruses are used as a system which is under expansion for the manufacture of plant-derived biopharmaceuticals. Transgenic plants are cheap and effective platforms for bulk vaccine and enzyme production (Hefferon, 2012; Figure 1). Plant viral expression vectors provide a reliable alternative for high levels of protein production *in planta* by carrying in its construct the epitopes for vaccines and full therapeutic proteins within its tissues. This offers a good prospect for the biomanufacture of important vaccines through proper biocontainment for critical diseases and offers a candidate for urgent protein therapeutics development in case of pandemics.

The deconstructed vector of BeYDV has been used for *Staphylococcus* enterotoxin B (SEB) vaccine production, which is considered as a tentative bio-warfare agent. The viral genes for coat and movement proteins have been deleted, and an expression cassette for a protein of interest has been inserted into BeYDV-derived expression vectors. When the geminiviral vector is delivered to leaf cells through *Agrobacterium*, it creates a large amount of recombinant DNA that may be used as a transcription template, resulting in a large amount of mRNA for the protein of interest (Chen et al., 2011). Antigens derivative from the *Hepatitis B virus*, HIV, HPV, and *NV* have also been successfully made (Hefferon and Fan, 2004; Huang et al., 2009; Regnard et al., 2010; Figure 1). The deadly *Ebola virus*, which was a huge life-altering epidemic causing huge economic and socio-cultural disruption in Africa, was similarly combated by utilizing monoclonal antibodies prepared using this technique by eliminating the production of hetero-oligomeric proteins (Huang et al., 2010). From a single-vector replicon, monoclonal antibodies against the *Ebola virus* (EV) GP1 protein and the *West Nile Virus* (WNV) E protein were produced and accumulated at levels of 0.23 to 0.27 mg/g leaf fresh weight in lettuce plants. A curtovirus, namely, the BCTV, was deconstructed to develop an exogenous protein production system which worked efficiently on addition of a virus-based suppressor of RNA silencing plasmid (Kim et al., 2007). The *Cassava vein mosaic virus* (CsVMV) promoter was used instead of the CAMV 35S promoter to create this vector. When the P19 gene-silencing suppressor was added, reporter gene expression rose by 320% at the RNA level and by 240% at the protein level. In *N. benthamiana*, the capsid protein of the *Hepatitis A virus* (HAV VP1) was linked to an Fc antibody fragment and expressed. After intraperitoneal vaccination, recombinant HAV VP1-Fc isolated by affinity chromatography was able to induce a serum IgG response. IFN- and IL-4 levels were also shown to rise after vaccination. The TYDV system, which offered a fresh twist to the mastrevirus-based geminiviral vector system, is made up of two expression cassettes: one encodes Rep/RepA under the control of the AlcA:AlcR promoter, and the other includes the gene of interest activated under the control of an ethanol inducible promoter, which may be triggered by simply spraying an ethanol solution on it. The gene of interest is broken into two sections, separated by a synthetic intron, and put into the INPACT (In Plant Activation) cassette. In this method, the gene of interest could only be translated from replicons created during geminivirus sequence activation and applied to remove the intron (Dugdale et al., 2013). This method has proved to be adaptable to a variety of host plant species, giving it a distinct edge over many other plant virus expression systems now on the market. The therapeutic protein vitronectin was generated and easily isolated from leaves with this virus expression system and sprayed with 1% ethanol as a proof of concept. A list of vaccines derived from geminiviral vectors is shown in Table 2.

**TABLE 2:** Therapeutic products and vaccines produced by geminiviral vectors.

Therapeutic protein	Viral vector	Host plant	Immunogenicity	References
SEB	BeYDV	*N. benthamiana*	+	Shen and Hohn, 1995
Norwalk virus VLPs	BeYDV	Tobacco, lettuce	+	Lai et al., 2012
WNV E protein Mab Ebola	BeYDV	*N. benthamiana*	−	Huang et al., 2009
Virus GP1 Mab	BeYDV	Tobacco, lettuce	+	Phoolcharoen et al., 2011
HPV-1 L1 HIV-1 type	BeYDV	Tobacco, lettuce	+	Phoolcharoen et al., 2011
C p24	BeYDV, mild	*N. benthamiana*	−	Regnard et al., 2010
HAV VP1	BeYDV, mild	*N. benthamiana*	−	Regnard et al., 2010
Vitronectin	BCTV	*N. benthamiana*	−	Chung et al., 2011
	TYDV	*N. benthamiana*	N/A	Dugdale et al., 2014

The easy scalability along with flexibility makes geminiviruses an attractive candidate for biomedical interventions in terms of vaccine production. This will provide innovative approaches ranging from developing modern, customized drugs to combating potential pandemics, and of course protecting the world’s poor from preventable infectious diseases.

## Concluding Perspectives

Plant viruses are usually seen mainly as disease-causing agents, which is only one part of the story. Through the decades of research, there has been an unraveling of a host of possibilities in the role elucidation of these pathogens. As we have extensively discussed in this review, geminiviruses and many other classes of viruses offer tremendous functional potential in genome editing, changing the way virus-induced agricultural havoc is generally tackled. Crops with a complex genome like wheat and recalcitrant crops like cassava and chili pepper have been successfully transformed using the aid of modified viruses, thereby opening immense prospects of engineering viral vectors for host genome improvements. Discovering new genes and their characteristics is also possible due to the rapid assays the transient GIGS procedures can present, making it a novel and pertinent molecular tool, bypassing the time taken for stable transformation protocols.

The past 30 years have been watershed for gemini-vector design, for applications in plant improvement. Geminiviral vectors have been used in multiple processes like ncDNA expression, vaccine and antibody production, gene silencing systems and expression systems, and CRISPR/Cas9 system construction for rapid assays, in the area of functional genomics. The biopharmaceutical industry has also realized the potential for gemini-vector utilization for vaccine development, where several vaccines are in clinical trials, and few have been approved in the past. The fluidity of geminiviral vectors into morphing successfully on any given “omics” aspect makes it a much-undervalued system of study in the current times, although apprehensions seem to arise due to possible geminiviral vector-linked environmental concerns, even though plant viruses have never posed any threat to human health as consumption of infected food has been done from centuries, albeit unknowingly.

An important perspective in genome manipulation is the identification of elements which could help in the development of geminiviral vectors for stable transgenics development. The current method extensively uses *Agrobacterium*-mediated delivery of the gene of interest cloned in non-viral vectors, which then involves a lengthy plant transformation procedure and subsequent generation of stable lines. As rapid development in sciences also deal with identification of shorter yet effective experimental strategies, geminiviruses can be exploited as a model virus for shorter infiltration strategy development which could be a prospective replacement to the *Agrobacterium* transformation stages with direct mechanical inoculation plant transformation. Therefore, gemini-vector construction–deconstruction with host genome integrative sequences should be exploited aggressively for mimicking *Agrobacterium*-mediated stable line generation.

Another significant concept that should be delved into is the overexpression and silencing of small RNAs through geminiviral vectors within the plant host genome. A notable disadvantage with geminiviral vectors is that it can only accommodate smaller sequences coding for size-restricted proteins as recombinant vectors with larger sequences are not stable. However, this can be advantageous in that it could serve as the backbone for delivering small RNAs in plants, thereby helping in modulation of regulatory processes in the DNA/RNA level, modulation of unwanted characters, and targeted overproduction of desirable traits in plants. Further, under the control of a suitable promoter, these vectors can be used as vehicles for overexpression of small peptides for many applications, such as peptide antibiotic nicin. Such viral vectors can be very useful for expressing venom peptides, having applications for developing anti-venoms, like mite allergens (Hsu et al., 2004; Kang et al., 2016). An in-depth investigation of roles is required to completely understand and realize the true potential of gemini vectors for the betterment of crop health and for bio-pharmaceutical role mining so that the middle- and low-income countries too can benefit from the end products.

## Author Contributions

BB and VH planned and designed the review. BB drafted the manuscript and prepared the table and figure. VH edited the manuscript. Both authors read and approved the final version of the manuscript.

## Conflict of Interest

The authors declare that the research was conducted in the absence of any commercial or financial relationships that could be construed as a potential conflict of interest.

## Publisher’s Note

All claims expressed in this article are solely those of the authors and do not necessarily represent those of their affiliated organizations, or those of the publisher, the editors and the reviewers. Any product that may be evaluated in this article, or claim that may be made by its manufacturer, is not guaranteed or endorsed by the publisher.
